# Electrochemical characteristics of microelectrode designed for electrical stimulation

**DOI:** 10.1186/s12938-019-0704-8

**Published:** 2019-08-01

**Authors:** Hongyan Cui, Xiaobo Xie, Shengpu Xu, Leanne L. H. Chan, Yong Hu

**Affiliations:** 1 Institute of Biomedical Engineering, Chinese Academy of Medical Sciences and Peking Union Medical College, No. 236 Baidi Road, Nankai District, Tianjin, 300192 China; 2 Department of Orthopaedics and Traumatology, The University of Hong Kong, 12 Sandy Bay Road, Pokfulam, Hong Kong China; 3 Department of Electronic Engineering, City University of Hong Kong, Tat Chee Avenue, Kowloon, Hong Kong China

**Keywords:** Microelectrode array, Parylene-C, Electrochemical characteristics

## Abstract

**Background:**

Microelectrode arrays play an important role in prosthetic implants for neural signal recording or applying electrical pulses stimulation to target nerve system. Safety and long-term reliability are essential requirements for microelectrode arrays applied in electrical stimulation. In design and fabrication of the microelectrode array, soft materials are generally chosen to be the substrate for the aim of achieving better compliance with the surrounding tissue while maintaining minimal damage. By flexing of the array to the surface, the array is capable of keeping a more stable electrical contact resulting in a significantly improved signal detected.

**Methods:**

In this study, we design and fabricate a flexible microelectrode array with gold as the electrode material and parylene-C as the substrate. The fabrication process of the array is presented. The in vitro electrochemical characteristics of the microelectrode are investigated by electrochemical impedance spectroscopy and cyclic voltammetry in a three-electrode electrochemical cell containing phosphate-buffered saline. Charge injection capacity measurements are carried out by multichannel systems and the CSC of the microarray is calculated.

**Results:**

Electrochemical results showed that impedance decreased with frequency. The average impedance of the Au electrodes at 1 kHz was 36.54 ± 0.88 kΩ. The average phase angle at 1 kHz was − 73.52 ± 1.3°, and the CIC of the microelectrode was 22.3 µC/cm^2^. The results demonstrated that the microelectrode array performed as expected for neuronal signal recording or stimulation.

**Conclusions:**

With parylene-C as the substrate, the microarray has good flexibility. The electrochemical characteristics’ results show that the array has the ability to resist any corrosion on metal–electrolyte interface and has good biocompatibility. This low-cost, flexible parylene-based, gold microelectrode array shows potential for use in implant neurological signal acquisition or neurostimulation applications.

## Background

Retinal degeneration characterized by loss of photoreceptors, including conditions such as retinitis pigmentosa (RP) and age-related macular degeneration (AMD), affects millions of people worldwide [1–4]. Development of human retinal prostheses to restore vision brings hope to individuals suffering from outer retinal diseases [5–8]. RP and AMD patients lose vision mainly because photoreceptors are damaged or degenerated. The two kinds of photoreceptor cell, rod and cone cells, exhibit different degrees of degeneration. The concept of prosthetic vision is that electronic components are used to convert light into an electrical signal that stimulates neurons in the visual pathway. The neural signal is then processed by the brain to generate phosphenes (i.e., flashes of light). In practice, the realization of prosthetic vision has proven complex and challenging [9]. Also, the success of a retinal prosthesis depends on several issues, efficient capturing of the visual images from the outside, transduction of the captured images into meaningful neurological signals, and subsequent activation of the residual inner retina (ganglion cells), from where visual information can be relayed to the visual cortex by the optic nerve. In the early stage of degeneration when retinal ganglion cells are spared, transmission of electrical signals from the retina to the brain by electrical stimulation is possible. In this process, the surviving retinal ganglion cells are electrically stimulated, transmitting signals to the visual cortex through the optic nerve, and then the visual image is integrated in the brain.

Apart from retinal stimulation, electrical stimulation is used in several neuro-prosthetic approaches. Platinum–iridium alloy has excellent mechanical properties and resistance to corrosion; the feature fits very satisfactory bio-engineering cable; the redox performance of platinum and Pt–Ir electrodes in saline allows artificial simulation of nerves for long periods. It has been used as a biometric sensor inside the cochlea [10]. Activated iridium oxide film (AIROF) and platinum black are always used as electrode materials, and the latter was chosen for recording AEPs from the rat brain. The polyimide-based microelectrode array proved to be capable of recording AEPs from rat cortex with reasonable amplitudes, when platinum black was chosen as an electrode material [11]. In a previous study [12], an intracochlear sound sensor-electrode system consisting of an intracochlear sound sensor (ISS) and a 50 μm Pt–Ir wire electrode was fabricated and tested. The system could sense acoustic signals and transmit electrical stimuli inside the cochlear, and it has potential applications including acting as the front end of a cochlear implant to treat sensorineural deafness or as a transducer in cochlear mechanics experiments.

Bioelectrode technology of flexible thin-film microelectrode arrays based on microelectro-mechanical systems (MEMS) enhances the development of epiretinal prosthetic implants and it has progressed rapidly [13–17]. Neural stimulation microelectrodes with diameters from 50 to 500 µm have been investigated in previous studies [18, 19]. In the application aspect, high resolution of the visual prostheses is desirable. However, high resolution means higher density and smaller size of the electrode, this also means higher charge density to activate neural response, while that may cause tissue damage due to the heat of the surrounding tissue, and also the signal to the visual system would generate higher power. In addition to the usual biomaterial issues such as toxicity, tissue encapsulation and cellular or immune responses that might be incited by the foreign materials, an electrical prosthesis must also provide long-term stability of the metal electrodes, while minimizing any tissue damage that occurs as a result of the electrical stimulation. Induced tissue damage will reduce the excitability of the tissue and limit the potential for vision restoration [9]. The microelectrodes need to be biocompatible and suitable for long-term implantation. Platinum (Pt) is the most commonly used electrode material because of its low impedance and high charge storage capacity [20, 21]. However, a long-term (42 days) stability test revealed that gold (Au) electrodes show a higher stability of capacitive behavior to reversible charge than Pt electrodes [22].

The selection of electrode materials for use in retinal prostheses requires consideration of biocompatibility, conductivity, and corrosion resistance. Parylene-C is often used as a substrate material because of its excellent combination of barrier properties (moisture barrier) and biocompatibility. Parylene-C [23] is widely used as a coating for many chronic implants for the human body such as stents, defibrillators, and pacemakers.

In our study, a flexible microelectrode array is designed with Au as the electrode material and parylene-C as the substrate. The electrochemical characteristics of the microelectrode array were investigated. We examined this array as a possible material combination for neural stimulation applications.

## Methods

### Materials

Parylene-C outperforms other substrate materials in terms of its dielectric constant, dielectric loss, water absorption, tensile strength, and Young’s modulus [24]. As shown in Table 1, parylene-C is superior to its counterpart, Parylane N in Mechanical and electrical properties. Parylene-C also tolerates room temperature chemical vapor deposition. Its low water permeability is suitable for long-term implantation.

**Table 1:** Properties of Parylane C compared to that of its counterpart Parylane N [25]

Properties	Parylene-N	Parylene-C
Typical mechanical properties		
Tensile strength (psi)	6500	10,000
Tensile strength (MPa)	45	69
Yield strength (psi)	6300	8000
Tensile strength (MPa)	43	55
Tensile modulus (Mpa)	2400	3200
Elongation at break (%)	40	200
Yield elongation (%)	2.5	2.9
Density (g/cm^3^)	1.11	1.289
Coefficient of friction		
Static	0.25	0.29
Dynamic	0.25	0.29
Water absorption (%, 24 h)	0.01 (0.019”)	0.06 (0.029”)
Typical electrical properties		
Dielectric strength, short time (Volts/mil at 1 mil)	7000	6800
Volume resistivity 23 °C, 50% RH (Ohm-cm)	1 × 10^17^	6 × 10^16^
Surface resistivity, 23 °C, 50% RH (Ohm)	10^15^	10^15^
Dielectric constant (Hz)		
60	2.65	3.15
1000	2.65	3.1
1,000,000	2.65	2.95
Dissipation factor (Hz)		
60	2E−04	0.02
1000	2E−04	0.019
1,000,000	2E−04	0.013

### Microelectrodes layout

**Fig. 1:**
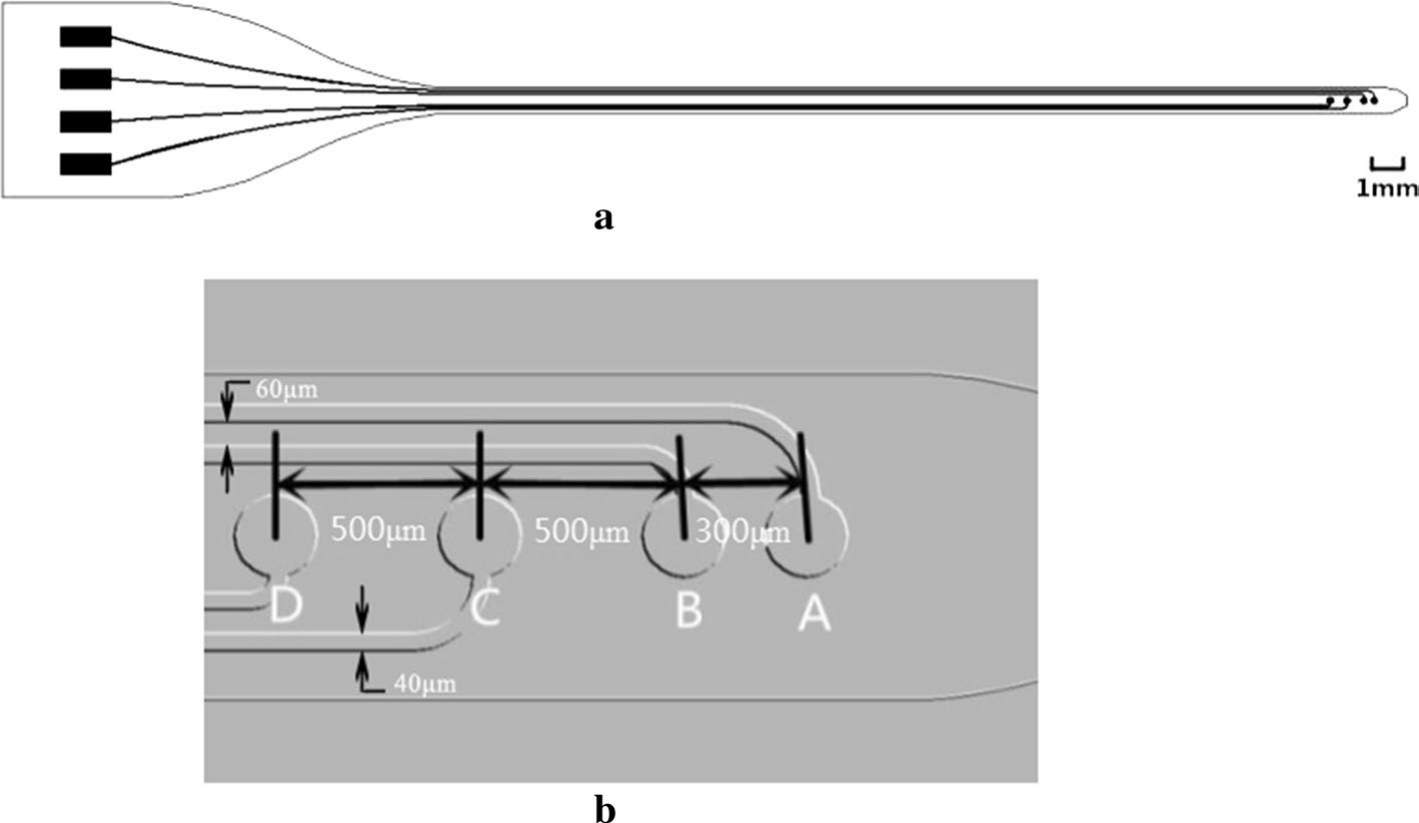
Diagrams of the microelectrode array. **a** Schematic diagram of the microelectrode array, **b** enlarged diagram of the tip of the array with four electrode sites

In this study, microelectrodes with a diameter of 200 μm were used [26]. The flexible microelectrode array based on parylene-C has four microelectrode sites that are arranged in a line, as illustrated in Fig. 1. The pitches between two adjacent electrodes are shown in Fig. 1b. The width of the interconnecting traces is 40 μm and the minimum distance between the interconnecting traces is 60 μm.

### Fabrication process

**Fig. 2:**
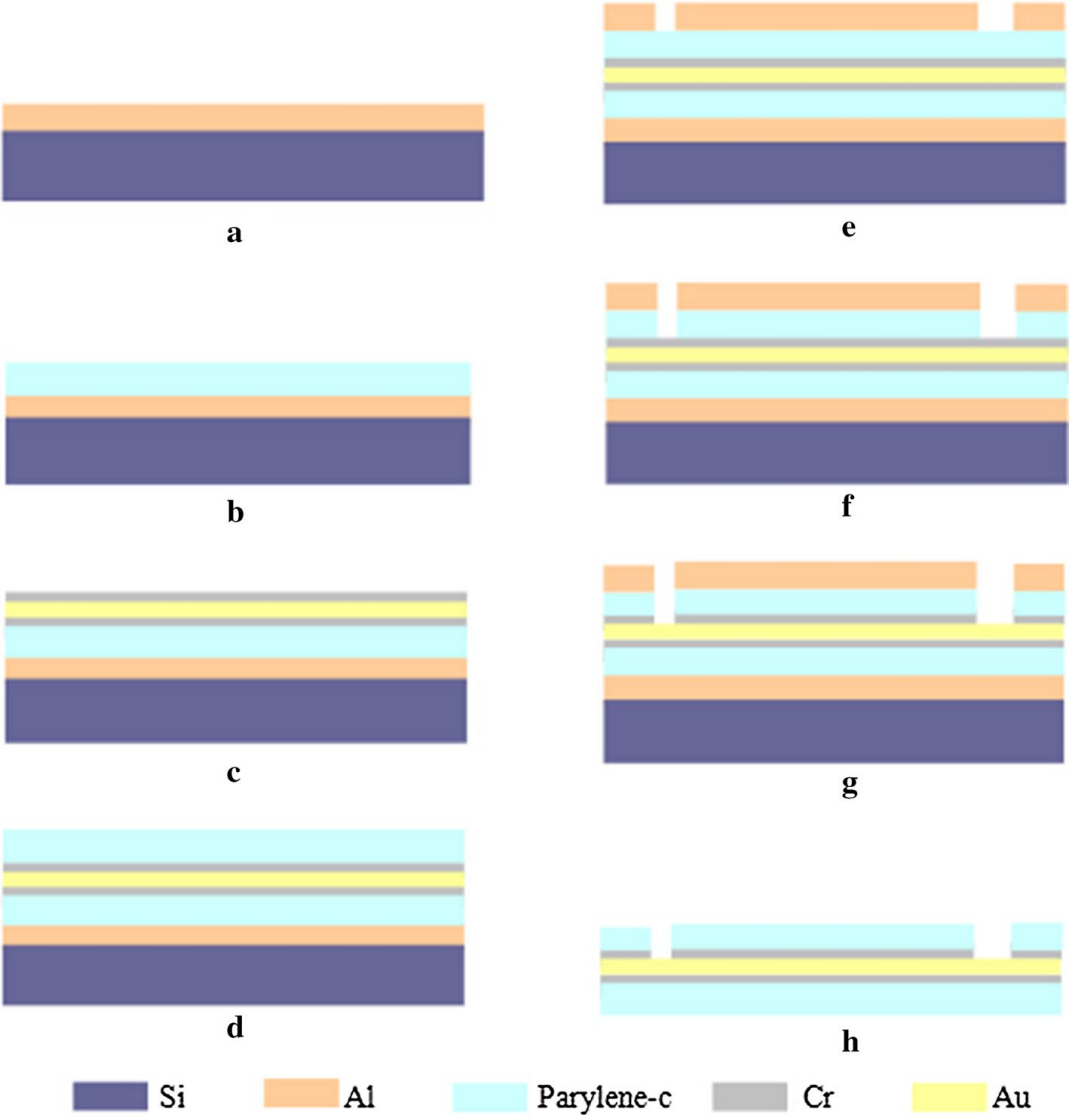
Fabrication process flow for parylene-based microelectrode array. **a** Evaporation of aluminum sacrificial layer onto the silicon wafer, **b** parylene-C as flexible substrate, **c** Cr/Au/Cr as the electrodes, metal wires and connecting pads, **d** parylene-C layer as the insulator layer, **e** Al layer added as a masking layer, **f** parylene-C processed with plasma dry etching, **g** photolithography and lift-off process for drain and source electrodes, **h** microelectrode array released from the silicon substrate

The microelectrode array based on parylene-C was fabricated using a similar procedure to that employed for a polyimide-based microelectrode array [21]. The fabrication process of the array is presented in Fig. 2. The array was assembled on a silicon wafer coated with a 300-nm-thick aluminum (Al) sacrificial layer to release the structure after fabrication. A 12- to 14-µm-thick parylene-C layer was deposited onto the silicon wafer as an insulating layer after salinization to enhance adhesiveness. Cr/Au/Cr (70/200/70 nm) metal layers were then patterned by sputtering and lithography to form electrodes, metal wires, and connecting pads. Cr was coated on Au to increase the adhesion of gold and insulating parylene layer. The array was then coated with an upper 12- to 14-µm-thick insulating parylene-C layer. A 500-nm-thick Al layer was deposited as a masking layer by evaporation, lithography, and electrochemical erosion. The upper parylene-C layer was processed by O_2_ plasma dry etching. After the masking layer was removed and Cr coated on the surface of the electrodes and connecting pads corroded, the array was released from the silicon substrate by electrolysis of the sacrificial Al layer. The surface of the microelectrodes of gold layer was exposed as shown in Fig. 2h.

### Electrochemical measurements in vitro

**Fig. 3:**
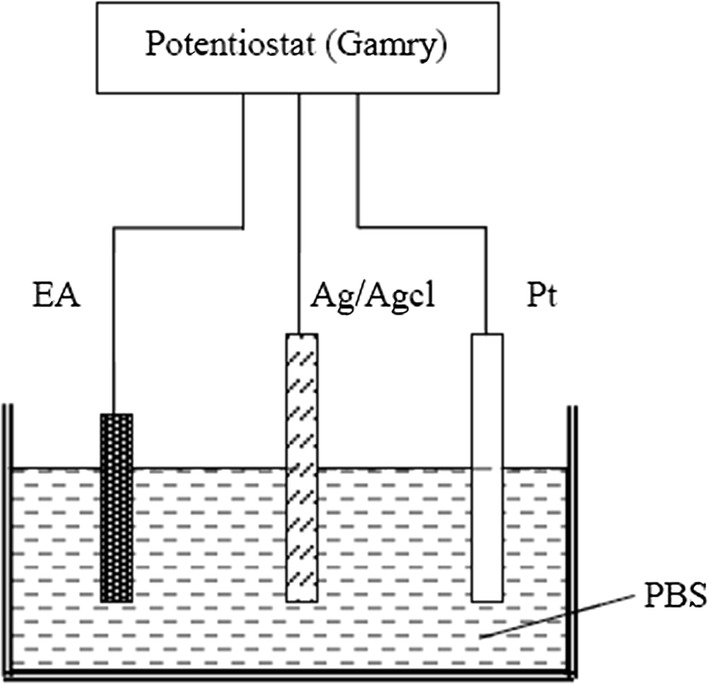
Diagram of three-electrode electrochemical system. EA is the microelectrode array under test

Electrochemical impedance spectroscopy (EIS) and cyclic voltammetry (CV) experiments were performed in a three-electrode electrochemical cell containing an Ag/AgCl reference electrode, Pt counter electrode, and an Au microelectrode immersed in phosphate-buffered saline (PBS) at pH 7.4 (Fig. 3). An AC voltage of 50 mV was applied using a potentiostat (Reference 600; Gamry Instruments, Warminster, PA, USA). During the test, a Faray shield was used to surround the electrode to be tested, with all parts of the shield electrically connected. The Faraday shield was electrically connected to the Reference 600+’s floating-ground terminal, and an additional connection of both the shield and the Reference 600+ floating ground to an earth ground. All data were collected at room temperature. Mean and variance method was applied to conduct statistic analysis of impedance and phase testing results. Statistical analysis was performed via Excel statistical data analysis.

### Charge injection capacity measurements

Based on the result of previous studies [27], activating thresholds of different electrode sizes up to 200 μm were tested and recorded; according to the threshold current, we calculated the average current density in Table 2.

**Table 2:** The average threshold for different electrode diameter up to 200 μm is T (μA) ± standard error of the mean (SEM). The average current density is CD (mA/cm^2^) ± SEM

Electrode diameter (μm)	Pulse width					
	60 μs		400 μs		1000 μs	
	T ± SEM	CD ± SEM	T ± SEM	CD ± SEM	T ± SEM	CD ± SEM
10	6.9 ± 0.6	87.90 ± 7.64	1.05 ± 0.04	13.38 ± 0.51	0.87 ± 0.03	11.09 ± 0.38
30	6.0 ± 0.4	8.49 ± 0.57	1.84 ± 0.09	2.60 ± 0.08	1.32 ± 0.04	1.87 ± 0.06
60	21.7 ± 0.5	7.68 ± 0.18	3.20 ± 0.20	1.13 ± 0.07	3.20 ± 0.10	1.13 ± 0.04
200	13.5 ± 0.4	0.43 ± 0.01	3.3 ± 0.10	0.11 ± 0.00	2.33 ± 0.07	0.07 ± 0.00

The charge injection capacity (CIC) of the Au microelectrode was measured using a cathodic-first, charge-balanced, biphasic, and symmetric current pulse applied by a four-channel, general-purpose, stimulus generator (Multichannel Systems, STG4004, MCS GmbH, Germany) with a pulse duration of 1 ms [26]. Current amplitude pulses were increased from 1 to 7 μA. The voltage responses of the microelectrode as a function of current amplitude were recorded with an oscilloscope. The experiments were performed in PBS (pH = 7.4) at room temperature using a two-electrode configuration with an Ag/AgCl reference electrode.

## Results

**Fig. 4:**
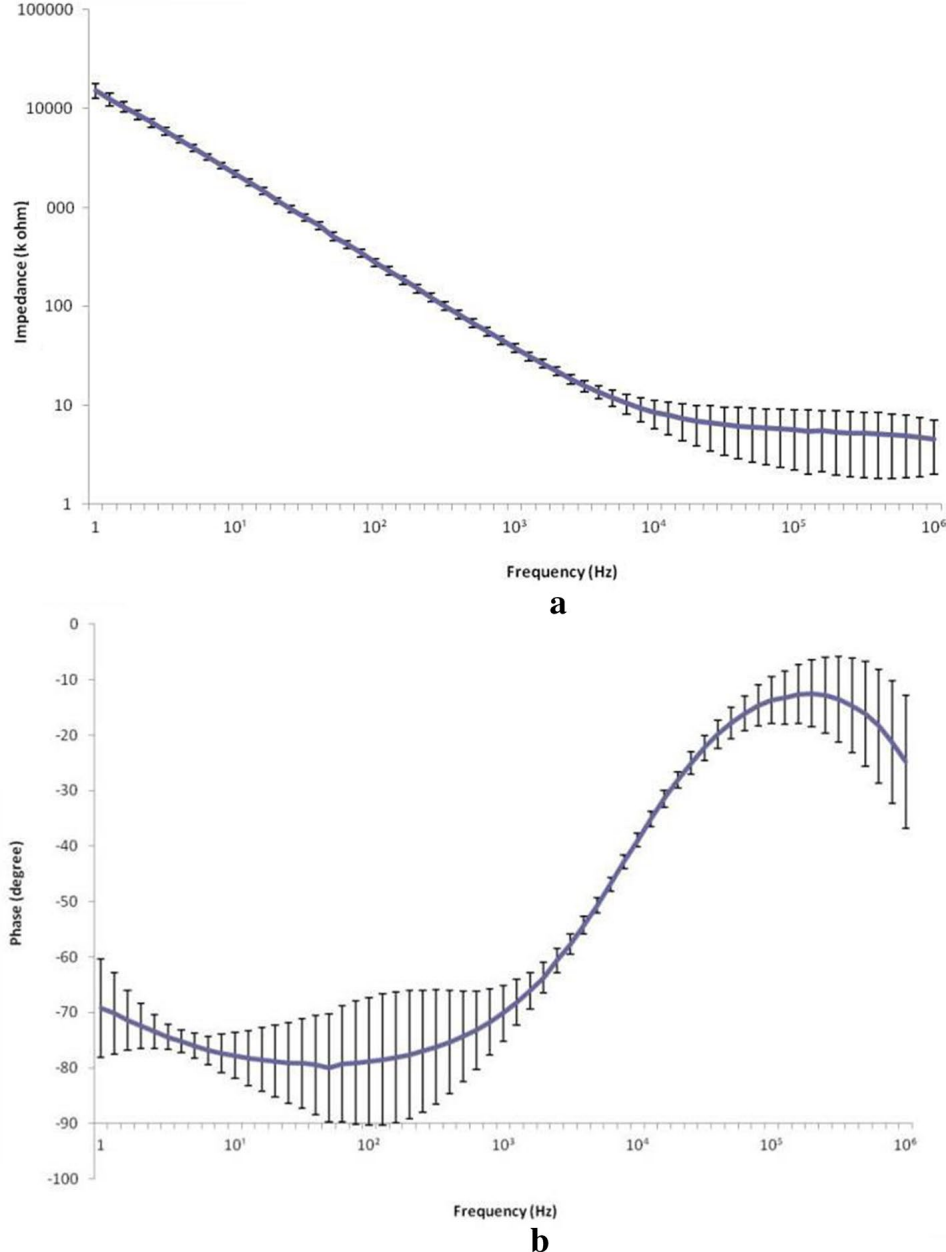
Average electrochemical impedance spectroscopy (EIS). **a** Average impedance, **b** Average phase. It shows the variation of average impedances and phases with frequency, respectively. Bars indicate standard deviation

**Fig. 5:**
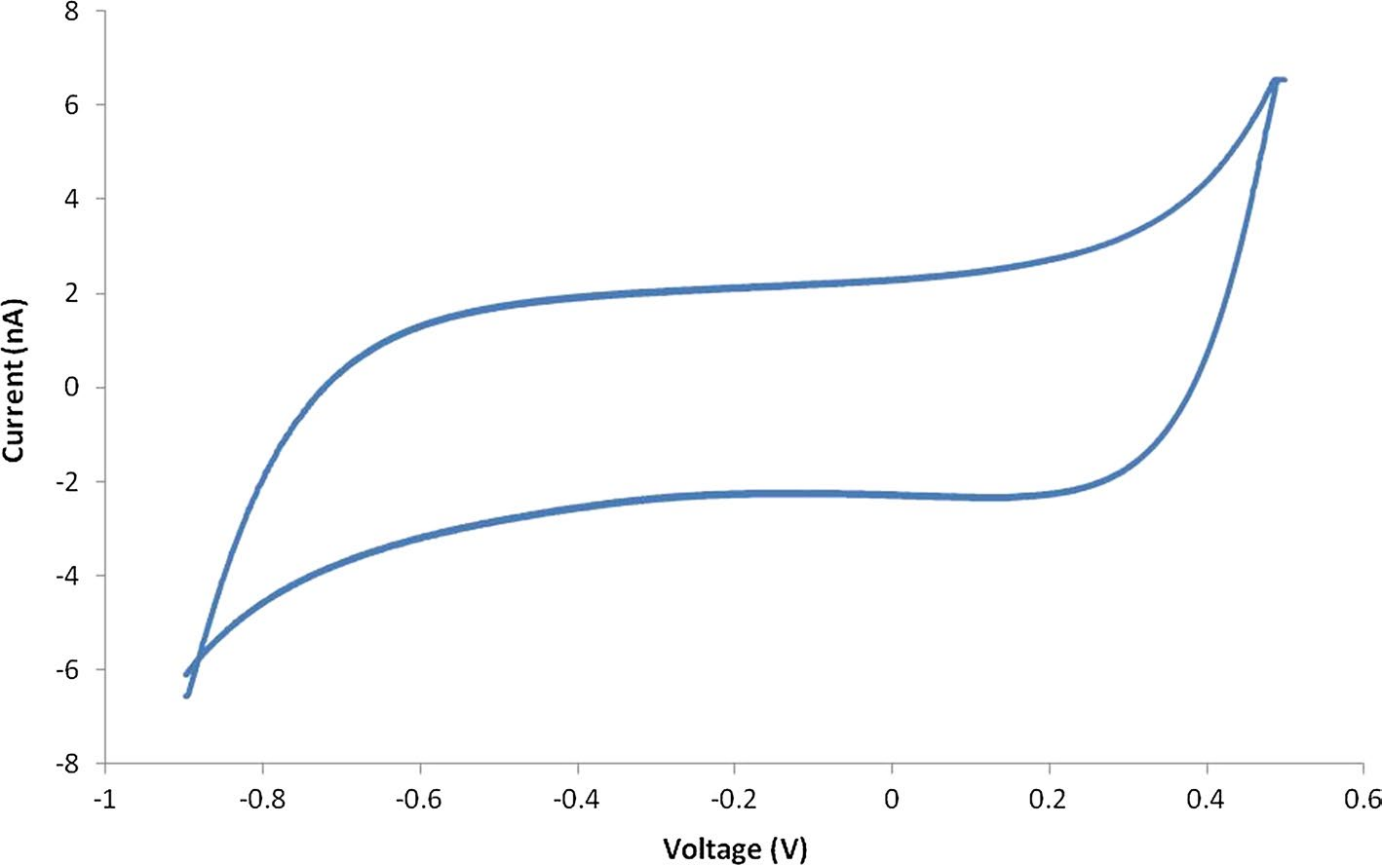
Cyclic voltammogram measured in PBS solution (pH = 7.4) at room temperature

Electrochemical characteristics were measured in the designed electrode as shown in Fig. 1. EIS data obtained for the microelectrode array are shown in Fig. 4. Impedance decreased with frequency. The average impedance of the Au electrodes at 1 kHz was 36.54 ± 0.88 kΩ. The average phase angle at 1 kHz was − 73.52 ± 1.3°. CVs recorded for the microarray at a sweep rate of 100 mV/s are presented in Fig. 5. The area enclosed by the CVs represents the charge storage capacity (CSC) of the microarray. The CSC of the microarray was calculated by dividing the total cathodic charge, i.e., the time integral of the cathodic current, by the scan rate. The average CSC of the Au electrodes was 103.33 ± 15 µC/cm^2^.

**Fig. 6:**
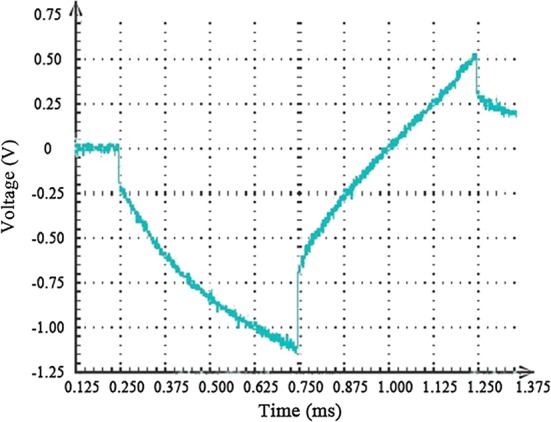
Voltage response of gold microelectrode to a biphasic and symmetric current pulse. Here, pulse width is 1 ms with amplitude I = 7 μA

The CIC is the maximum transferred charge of the microelectrodes before irreversible reactions occur. The CIC of the microarray was calculated as the product of current and pulse width per unit area. Figure 6 shows a representative potential voltage response to applied current pulses recorded from one of the gold microelectrodes. The critical current was 7 µA when the potential cathode voltage reached 0.55 V. The CIC of the microelectrode was 22.3 µC/cm^2^.

## Discussion

Biocompatibility of the materials, density of electrode sites, and electrochemical characteristics are the important design parameters for microelectrode array. We designed, fabricated, and tested a flexible microelectrode array with parylene-C as the substrate, Au as the microelectrode material, and different pitch intervals between the electrode sites. The in vitro electrochemical characteristics of the array suggest its feasibility for use as a retinal implant. The manufacturing process is simple and economical.

In the design of neural stimulation electrode, a low electrode–electrolyte impedance interface is critical, and a complete understanding of the physical processes contributing to the impedance is needed in the design of a low impedance interface [28]. Equivalent circuit models have long been used to model the interface impedance. Early in 1899 [29], it first proposed that the interface could be represented by a polarization resistance in series with a polarization capacitor. Later research results revealed that the polarization capacitance exhibited a frequency dependency leading to the introduction of Fricke’s law [30], and the use of a constant phase angle impedance to represent the impedance of the interface capacitance. Later work with rapid electrode reaction systems resulted in the well-known Randles model, consisting of an interface capacitance shunted by a reaction impedance in series with the solution resistance [31]. In previous research [28], an electric model was used to describe the physical processes and extended to quantify the effect of organic coatings and incubation time, and electrochemical impedance spectroscopy (EIS) has been used to electrically characterize the interface for various electrode materials, and the results demonstrate the benefits of using this model to better understand the physical processes occurring at the interface in more complex, biomedically relevant situations.

Development of optimal active neural prostheses that can monitor the physiological state of neural tissue has been investigated for decades. In neuro-prosthetic study, impedance characterization of the electrode–electrolyte interface is of paramount importance in the fields of impedance-based, neuroprotheses, and in vitro communication with electrogenic cells [28]. A high impedance would result in a large applied electrode voltage leading to undesirable electrochemical reactions that may be harmful to cellular cultures. On the recording side, the extracellular signals are low, on the order of microvolts for neurons. The neural signals will be lost in the noisy, ion-based electric fluctuations of the surrounding electrolyte media if the electrode impedance is not low enough. A well-characterized, fully understood interface impedance leads to an optimized electrode–electrolyte interface design. Neuroprotheses, and in particular cochlear implants, represent an important application of impedance characterization. The current applied to stimulate hearing via a cochlear implant is determined from the known electrode impedance [32], which is designed to be as low as possible to avoid cell damage [33]. Neural prostheses use charge recovery mechanisms to ensure the electrical stimulus is charge balanced. Nucleus cochlear implants reduce all stimulating electrodes between pulses in order to achieve charge balance, resulting in a small residual direct current (DC) [32]. Jiang et al. [22] implanted Pt electrodes in the eyes of rabbits. Electrochemical impedance of a 200 µm-diameter electrode (Fig. 4b) in 0.1 M PBS electrolyte shows the phase angle expected for an Au stimulating electrode when compared with those found in the literature [21, 22]. The good high-pass characteristic of the array was consistent with other electrode impedances described in the literature [22]. The error bars in Fig. 4 represent the distribution of the data. As we can see the impedance of the electrode is quite stable with very little error bar, in the frequency range of 1–10 k Hz, and both impedance and phase value present stable trend in the frequency range of 1 k–10 kHz that falls in the interval of 0.1–1 ms, the pulse duration used for neural stimulation. Our microelectrode provided smaller size and lower impedance than that of Li [21]. Compared with Pt, Au electrodes are more cost effective. First, Au has long been used in the microfabrication industry and a series of reports could be found. This promises higher satisfactory rate of the array fabricated [34]. Second, mechanically, Pt is significantly stiffer than the neural tissue with which it interfaces. Electrode coatings, in particular polymeric films which utilize conductive polymersor hydrogels, have been shown to impart a softer electrode interface [35]. Third, in neural electrode field, with the increasing demand to reduce the size of electrodes, driven by the need to make smaller but higher resolution implants, Pt electrical properties have become a challenging issue. Pt, as a conventional neural electrodes material, its charge injection capacity (CIC) is limited below 0.15 mC/cm^2^ [20]. To increase the charge transfer capacity, a wide range of materials have been adopted as the coating layer [36]. Thus, the coating of the electrode must be taken into consideration when weighing the cost of fabrication and Rodger et al. [23] reported platinum or titanium–platinum microelectrodes based on parylene substrate. High charge injection capacity, flexibility and biocompatibility are key properties of microstimulation and neural applications [37]. During the manufacturing process, parylene-C was used as the substrate electrode material because of its good flexibility and biocompatibility [38, 39]. The CSC (103.33 ± 15 μC/cm^2^) as a parameter for qualitative comparison of various electrodes and electrode materials cannot provide quantitative data for stimulation protocols. For stimulation, the charge which can be actually injected during a stimulation pulse (charge injection capacity, CIC) determined by reactions contributed to the charge transfer during a single stimulation is smaller than the CSC [40]. As the electrode is implanted in the tissue, electrode–tissue interface, electrochemical reactions and heat production generally accompany charge injection. These factors constrain maximum charge injection levels and stimulation electrode area, which ultimately constrains the spatial resolution that could be achieved by electrical stimulation. The potential biocompatibility and long-term functional stability of a retinal prosthesis are further complicated by ongoing anatomical and physiological changes that inevitably occur within the retina in patients with retinitis pigmentosa [41]. Although the CIC of our Au microelectrode array is lower than those of the Pt microelectrode and the Pt–Ir microelectrode developed by Petrossians et al. [26], our array still meet the electrochemical requirements of visual implants. In earlier studies, McCreery et al. reported that the electric charge density to elicit an electrically evoked potential without damaging neural tissue was 10 µC/cm^2^ [42]. For accurate measurement of very small current, some methods have been applied to reduce the noise of the equipment effectively [43]. In the present study, Fig. 5 is the cyclic voltammogram of the array measured in PBS solution. The low level current was measured using a Faray box to shield the array and the test system to reduce the noise due to AC interference. Humayun and coworkers reported the charge densities of a bullfrog (2.98 µC/cm^2^), normal-sighted rabbit (8.92 µC/cm^2^), and rabbit with outer retinal degeneration (11.9 µC/cm^2^) as threshold stimulating charge densities [44, 45]. In this study, a flexible microelectrode array was designed with Au on the substrate of parylene-C. These charge densities are within the safe limit of our Au microelectrode array (22.3 µC/cm^2^).

In principle, the larger the electroactive surface area is, the lower the charge transfer resistance is [23]. A low electrochemical impedance (compared to the analog front end impedance) is required for the recording electrodes to achieve a high signal-to-noise ratio [46]. The average impedance of our electrodes at 1 kHz was 36.54 ± 0.88 kΩ at our designed electrode area of 0.04, This translates to an electrochemical impedance of 1.15 Ω/cm^2^, which was lower than that of standard gold of 10 Ω/cm^2^ [46]. These results indicate that our electrode is suitable for extracellular recordings of single or small populations of neurons recording. Moreover, the surface contact in three dimensions should be considered. In another word, the surface electroactive area is not the area in 2-D. The microelectrode’s surface by our fabrication process would increase a better contact by surface process, which may increase three-dimensional contacts to achieve lower impedance in a smaller area. Our electrodes also meet the compliance voltage. The stimulation current for a 1024-channel retina prosthesis is reported to be 30–300 μA [47]. The required compliance voltage of the current driver will, therefore, be up to 10.8 V (multiplying 300 μA by the impedance of 36.54 kΩ), which can be realized by circuit technology.

In the respect of eye implantation, the fact that retina is an exceptionally soft and fragile tissue increases the difficulty of epiretinal surgery and prosthesis implantation. An appropriate stiffness in the construction of the prosthesis is essential to aid the implantation during surgery so that the prosthesis will closely contact the ganglion layer and take the shape of retina without retinal compression. However, overly increasing the stiffness of the prosthesis will increase the mechanical pressure on the retina and may result in tissue damage. On the other hand, as a special neural implant, the microelectrodes should also be safe and acceptable for long-term use [22]. To design an ideal epiretinal microelectrode, considerations must be taken from biology, medicine, electrical and mechanical engineering, as well as the chemical properties of each component [48], to stimulate the retina and thereby the visual pathway via electrical stimulation. Some early subretinal designs used photodiodes as power sources and failed due to the lack of power. Later designs with energy supplied from external power sources report remarkable success [7].

During the process of implant, retinal tears and large retinal detachments can occur during surgery; placing the epiretinal devices on the surface of the retina can cause compression injury to the retina. Epiretinal devices secured with tacks over normal retinas are observed to show little histological change in the underlying retina. The presence of the implant in the eye can cause proliferation of fibrous tissue and its complications, such as tractional retinal detachment and retinal striae, both of which cause the electrodes to be lifted from the surface of retina. This interferes with the conduction of the current and, therefore, the functioning of the implant [49].

Rodger et al. designed arrays where the inflection points of interconnecting lines were at right angles [23]. As a result, the stress concentration at right angles increased the possibility of fracture during the fabrication process. In our array, the inflection angles of microelectrodes were modified to reduce local stress concentration, which successfully avoided fracture at the inflection point during manufacturing process. The curved angle design is based on the consideration of manufactory process and the insertion process of the electrode. The manufacturing of the electrode uses a “lift-off” processing technique [37]: a metallic film of Au was sputtered and patterned according to the layoff of the electrode, and the electrodes and bonding pads and the interconnecting lines are on this layer. Later, after the masking layer was removed and Cr coated on the surface of the electrodes and connecting pads corroded, the array was released from the substrate by electrolysis of the sacrificial Al layer. During the “lift-off” process, the inflection point of the connecting line, if designed as straight angle, delamination or fracture of the line is easy to occur due to the stress at the inflection point. Also, delamination issues were reported during neural stimulation, which could be due to poor adhesion between the coating material and the metal electrode [37]. The curved angle design will increase the contact area so as to strengthen the adhesion at the inflection point.

In the aspect of neural implantation, the sharp end of the right angle inflection point tends to cause damage of the surrounding neural tissue; the curved corners were utilized to reduce the neural damage resulting from the electrode array implantation [22]. Our design provides a wide range of flexibility of the microelectrode array that will aid implant surgery.

## Conclusions

In this study, we designed, fabricated and tested a low-cost microelectrode array using Au as the electrode materials and parylene-C as the substrate with various pitch distances. In vitro electrochemical results showed that the array meets the requirements for stimulating neural tissue in terms of low impedance and reasonable CIC. We provide a new solution for bioelectrode technology for its cost-effective manufacturing procedures, low impedances and good flexibility. It will greatly contribute to the development of visual prostheses.

## Data Availability

The datasets analyzed in this study are available from the corresponding author on reasonable request.

## Abbreviations

RP: retinitis pigmentosa
AMD: age-related macular degeneration
MEMS: microelectro-mechanical systems
EIS: electrochemical impedance spectroscopy
CV: cyclic voltammetry
PBS: phosphate-buffered saline
CIC: charge injection capacity
CSC: charge storage capacity
